# A Five-Bundle Intervention to Improve Blood Culture Use in a Tertiary Hospital in Romania

**DOI:** 10.3390/antibiotics13111040

**Published:** 2024-11-04

**Authors:** Alina-Ioana Popa, Daniela Tălăpan, Gabriel-Adrian Popescu

**Affiliations:** 1 Faculty of Medicine, “Carol Davila” University of Medicine and Pharmacy, 050474 Bucharest, Romania; dtalapan@yahoo.com (D.T.); gabrielp9@yahoo.com (G.-A.P.); 2 “Prof. Dr. Matei Balș” National Institute for Infectious Diseases, 021105 Bucharest, Romania

**Keywords:** blood culture collection, contamination rate, bacteremia diagnosis

## Abstract

**Objectives**: The aims of this study were to evaluate the efficacy of a five-bundle intervention and to decrease the number of cases in which only one set of blood cultures is collected prior to starting antimicrobials. **Methods**: The study group consisted of the two hospital wards that have the highest collection rate (120 blood cultures/1000 patient days and 121.4 blood cultures/1000 patient days, respectively), and the control group consisted of the other three adult wards. The collection protocol was changed, and a bundle of five measures was introduced: one-on-one discussions with the nurses, 2% chlorhexidine in 70% alcohol for disinfection, ensuring the use of sterile gloves, sterile wipes, a checklist for the materials needed, and a copy of the collection protocol. The impact of these changes was followed over a 5-month period. **Results**: Prior to the intervention, the contamination rate was higher in the control group (6.5%) versus the study group (4%), *p* = 0.00578. The before–after analysis revealed a significantly reduced contamination rate in the control group (4.6% vs. 6.5% *p* = 0.0099), but it was above the one obtained in the study group (3.1% vs. 4%, *p* = 0.1635). The number of infectious episodes in which one blood culture set was collected decreased significantly in the study group (77/311 vs. 139/456, *p* = 0.041). **Conclusions**: The intervention decreased the contamination rate and the number of infectious episodes in which one blood culture set is collected.

## 1. Introduction

Blood cultures are the gold standard for the diagnosis of bacteremia. They are included along with other tests in the first-hour bundle developed by the Surviving Sepsis Campaign [1] and should be collected prior to starting antimicrobial therapy, otherwise the chance to identify the etiologic agent decreases significantly. In an analysis performed by Cheng et al. [2], the percentage of positive blood cultures was significantly higher when collected prior to antimicrobial therapy initiation (31% positive blood cultures when collected before antimicrobial treatment vs. 19.4% positive blood cultures collected during the first 240 min after antimicrobial administration, *p* < 0.001).

Blood cultures can fail to retrieve the real etiologic agent of the infectious episode if they get contaminated. The most common contaminants are coagulase-negative (CoN)-staphylococci, *Corynebacterium* spp., *Micrococcus* spp., viridans group streptococci, *Cutibacterium* spp., and *Bacillus* spp. [3,4].

According to the published literature, the contamination rate should not exceed 3%, with a new goal of 1% set by the CLSI (Clinical and Laboratory Standards Institute) in 2022, when best practices are followed [5,6,7]. Blood culture contamination can lead to administration of unnecessary antimicrobial treatment with associated risks, such as *Clostridioides difficile* infection, antimicrobial resistance selection, and increased hospital length of stay [8,9]. A study published by Andrews et al. [10] in 1997 showed that every hospital admission day increases the risk for adverse events by 6%. Dempsey et al. [8] performed a systematic review of the economic costs associated with blood culture contamination. They evaluated 12 studies (10 performed in the US, one in Taiwan, and one in Northern Ireland) conducted during 1991–2014 and revealed that in 41% of these cases, antimicrobial therapy was prescribed in an unjustified manner. Vancomycin was the most frequent antimicrobial administered to the patients with contaminated blood cultures, being used in up to 59% of the reported cases. Besides the costs associated with the antimicrobial itself, secondary costs for assessing vancomycin blood levels were added. The attending physicians ordered secondary cultures for the patients with false-positive blood cultures. Overall, additional hospital costs in the setting of a contaminated blood culture was between USD 2923 and USD 5812 [8].

In order to reduce the contamination rate, several interventions were designed, and disinfectant agents were tested. These interventions included educational and training programs (PowerPoint presentations, live demonstrations, video materials for blood culture collection) and feedback from the staff collecting blood cultures [11,12,13]. Other interventions consisted in phlebotomy dedicated teams [13,14], the usage of blood collection diversion devices [15,16,17,18,19], sterile gloves [20], collection kits consisting of blood culture bottles, alcoholic chlorhexidine and disinfectant swabs, sterile fields, and in some cases a blood culture collection brochure or checklist [21,22,23]. All these interventions, either applied separately or associated, were successful in reducing the contamination rate.

Our objectives were to evaluate the efficacy of a bundle of interventions that do not involve high costs, in the settings of our hospital and medical system, as well as the persistence over time of any beneficial effects. We also aimed to decrease the number of cases in which a single set of blood cultures is collected before starting antimicrobial therapy.

## 2. Results

### 2.1. Before-Intervention Data

From January to July 2023, blood culture data (collection, contamination, and positivity rates) were analyzed. The data revealed that from the 3856 blood cultures collected, 224 were contaminated, leading to a hospital contamination rate of 5.8%.

During the pre-intervention period, the contamination rate in the study group was 4% (min; max: 2%; 5.6%), while in the control group, it was 6.5% (min; max: 4.2%; 9.3%), *p* = 0.00578.

The number of blood cultures/1000 patient days was higher in the study group (120.8/1000 patient days) than in the control group (103.5/1000 patient days), *p* < 0.00001.

In the study group, in 30.5% (139 sets/456 infectious episodes) of the infectious episodes, a single blood culture set was collected, while in the control group, this was observed in 42% (376 sets/895 infectious episodes) of the cases; the difference between the two groups is significant, *p* < 0.00001. The complete data regarding the number of blood cultures collected/infectious episodes are listed in Table 1.

From the 22 nurses that responded to the questionnaire, 76% answered correctly to the general questions related to blood cultures and their collection, but only 22.7% of them were familiar with the current collection protocol and thus adherent to it.

### 2.2. Post-Intervention Data

During August–December 2023, in our hospital, 2596 blood cultures were collected, and 132 sets were contaminated, leading to a contamination rate of 5.1%. The contamination rate was 3.1% (min; max: 0.9%; 4.3%) in the study group, while in the control group, it was 4.6% (min; max: 3%; 5.7%), *p* = 0.0559, and in the rest of the hospital wards, it was 8%. In the study group, one blood culture set/infectious episode was collected in 24.8% (77 sets/311 infectious episodes) of the cases, while in the control group, one blood culture set/infectious episode was collected in 39.9% of the cases (270 sets/677 infectious episodes), *p* < 0.0001. The complete data regarding the number of blood cultures collected/infectious episodes are listed in Table 1.

The number of blood cultures sets/1000 patient days was significantly higher in the study group (100/1000 patient days) than the ones in the control group (91.6/1000 patient days), *p* = 0.0466 (2 side).

### 2.3. Before–After Analysis

During January-July 2023, the contamination rate was significantly higher in the control group (6.5%) versus the study group (4%), *p* = 0.00578 (2-side). After the intervention, a decrease in the contamination rate was observed in both groups: study group 3.1% and control group 4.6%. The before–after comparison revealed a significantly reduced contamination rate in the control group (*p* = 0.0099), but it did not reach the recommended limit of 3% and it was also above the contamination rate obtained in the study group. Even though the reduction in the contamination rate in the study group was not significant (*p* = 0.1635), the contamination rate obtained was 3.1%, close to the previous target proposed. Also, when taken individually, the contamination rate was close to the recommended 3% for both wards included in the study group (WA = 3% and WB = 3.2%).

The number of infectious episodes in which only one blood culture set was collected decreased significantly in the study group (77/311 vs. 139/456, *p* = 0.041). The complete data can be found in Table 1.

**Figure 1:**
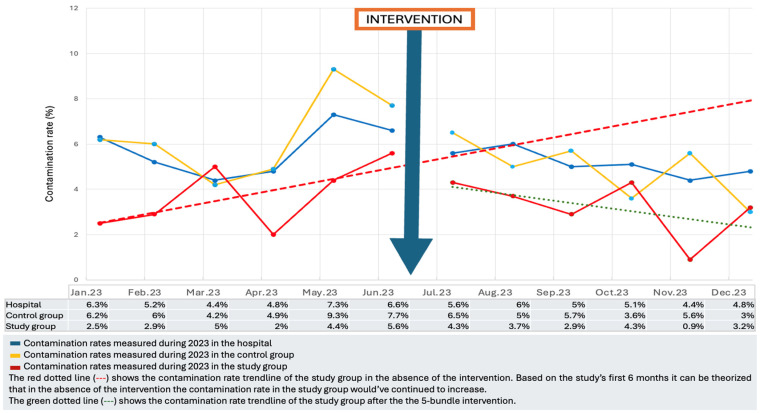
Evolution of the contamination rate during January–December 2023.

The complete data on the identified contamination rates and their evolution during 2023 can be found in Figure 1.

**Figure 2:**
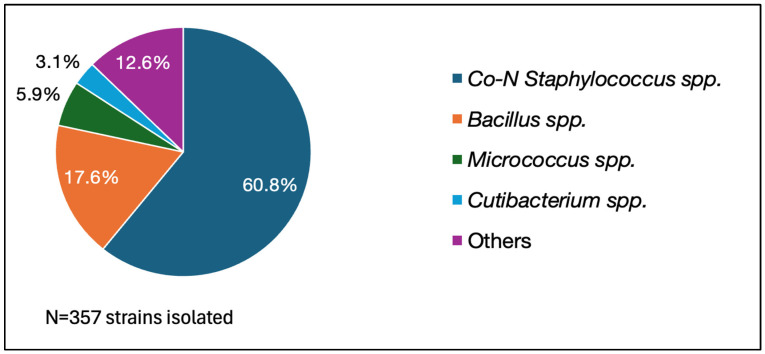
Distribution of the contaminants isolated from blood cultures in 2023.

**Figure 3:**
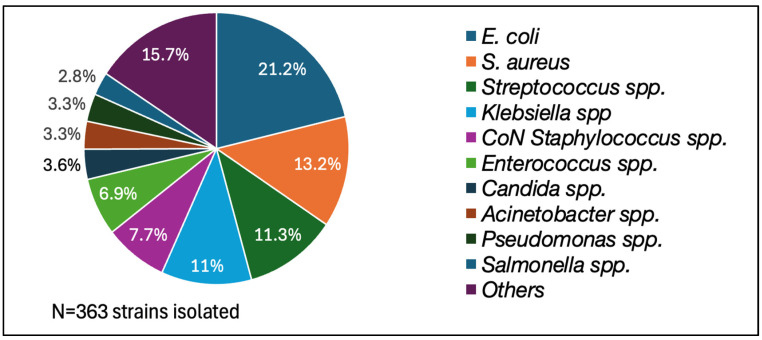
Distribution of the pathogens isolated from blood cultures in 2023.

Overall, in 2023, in our hospital, 6452 blood cultures were collected for 3174 infectious episodes, with one blood culture set/infectious episode collected in 47% of the cases (1491/3174 infectious episodes). The contamination rate obtained was 5.5% (356 sets/6452 sets), CoN-staphylococci being the most frequent contaminant (60.8%). The most frequent pathogens isolated were *E. coli* (21.2%), *Staphylococcus aureus* (13.2%), and *Streptococcus* spp. (11.3%). The complete data are presented in Figure 2 and Figure 3.

## 3. Discussion

Our hospital setting is different compared to other medical units because it treats only patients with infectious diseases. Thus, it would be expected for blood cultures to be sampled more frequently compared to other hospitals and the collection process to be carried out in the most appropriate manner (determined by the increased frequency of repeating the collection process).

Blood cultures can identify the etiologic agent and guide the antimicrobial treatment in patients with bacteremia. Their contamination can lead to a false diagnosis and the administration of an incorrect or unnecessary antimicrobial treatment that can favor *C. difficile* infection [8,9]. Also, it has been shown in studies performed by Dempsey et al. [8] and Alahmadi et al. [24] that contaminated blood cultures are associated with additional costs determined by unnecessary antimicrobial treatment administered in patients with contaminated cultures and increased length of stay. In this setting, it is important to find ways to limit the blood culture contamination phenomenon. In order to ensure the best practice when it comes to blood culture collection, target contamination rates were set in the USA. While the CLSI set a blood culture contamination target initially below 3% and later a best practice goal of 1% [5,6,7], in Europe there is no well-established objective in this manner. A major source for blood culture contamination is the patient’s skin flora [4]. Approximately 20% of bacteria are located in the deep structures of the skin where they are protected from the action of antiseptics by the lipid compounds synthesized by the sebaceous glands [25]. Thus, it was found that a high density of anaerobic bacteria is located next to the sebaceous glands, while aerobic bacteria, especially cutaneous staphylococci and corynebacteria, prefer anatomical regions poor in sebaceous glands [26]. In this context an alcohol-based antiseptic should be used first (because it acts as a degreaser). Caldeira et al. [27] performed a systematic review to determine the most suitable disinfectant for blood culture collection. They concluded that alcoholic chlorhexidine was superior to iodine povidone and that the association between alcohol and iodine povidone was not useful. When chlorhexidine alone was compared to tincture of iodine for the reduction in blood culture contamination, they were found equivalent [28]. When analyzing different skin antiseptics (alcoholic and non-alcoholic antiseptics, iodine compounds), Liu et al. [29] concluded that they have similar activity and that other factors, like different intervals between disinfection and the venous puncture and performance of those collecting blood cultures, may affect blood culture contamination rates.

After monitoring the evolution of the contamination rate in our hospital between January 2019 and July 2023, we identified a significantly higher contamination rate compared to the recommended limit of 3% [5,6,7]. Also, the degree of use of these tests was suboptimal, with a single set of blood cultures/infectious episodes collected in most cases. Before starting the intervention, it was decided to evaluate the nurses’ knowledge on blood cultures and to identify the reason behind the high contamination rate. The questionnaire answers revealed that the lack of knowledge on blood cultures was not the cause of the high contamination rate. The high contamination rate could be explained by other factors, such as the nurses’ exhaustion and their low adherence to the current collection protocol, as revealed by the questionnaire responses and the one-on-one discussions with the nurses. These findings were discussed with the heads of the two wards, and since the physicians ordered blood cultures, they were encouraged to recommend the collection of two blood culture sets initially. A PowerPoint presentation was developed to explain the new collection protocol and the reasons behind these changes. The material was presented during informal meetings. Previous disinfectants used (70% alcohol and iodine-povidone) were replaced with 2% chlorhexidine in 70% alcohol because it takes a short time to dry [30], it acts both as a degreaser and disinfectant agent, and it can be used in patients allergic to iodine. The analysis showed that nurses appreciated the shortening of the waiting period necessary for the skin to dry. Even though meta-analysis showed no superiority of alcoholic chlorhexidine over other disinfectants, it has been used as the primary disinfectant in a number of interventions [7,13,31]. Marcelino et al. [31] stated that the addition of chlorhexidine helped in reducing the contamination rate. The transition from a non-sterile collection protocol to a sterile protocol was made through the introduction of sterile gloves and sterile wipes. These measures were shown to reduce blood culture contamination [20].

After analyzing multiple examples of interventions for reducing blood culture contamination rates, we chose to build a package of measures that are among the most effective, achievable, and sustainable for our hospital, financially and through the participation of the medical staff. The intervention resulted in a significant increase in the initial blood cultures collected/infectious episodes, and it almost reached the target of 3% contamination in the study group. Although a reduction in the contamination rate was observed in the control group, its level remains higher than the one in the study group.

Other interventions that have proven to be effective in reducing the contamination rate are blood diversion devices [15,18,19] and phlebotomy teams [3,13]. The blood diversion technique allows the first milliliters of blood that might get contaminated with the skin flora to be diverted in a specific device, thus preventing contaminants from entering the blood culture bottles [16,17]. Simple sterile heparin tubes were also effective in reducing the contamination rate; in addition, they are less expensive, and the blood can be used for further investigations (ex., blood chemistry tests) [15,18,19]. Although effective, these measures are associated with additional costs.

Our bundle did not add any significant additional costs to the hospital since the materials already existed in the hospital. This offers an advantage over other measures for contamination reduction. Similar to other studies [7,13,31], this analysis found that major components in the success of this intervention were the informal meetings and feedback.

Similar to other published analyses [4,12,32], our study revealed that CoN-staphylococci were the most common contaminants isolated.

An important aspect that can dictate the efficacy of the blood cultures for establishing a diagnosis is the rate of true positive results. An analysis performed by Cockerill et al. [33] on over 10 000 blood cultures collected in patients without endocarditis revealed that the rate of positive results in adults is proportional to the quantity of blood collected, being significantly higher when 40 mL of blood were collected than when only 10 mL of blood were collected.

An objective of the current intervention was to decrease the number of cases in which only a single blood culture set was collected per infectious episode. Our intervention managed to decrease the number of cases in which only a single blood culture set was collected per infectious episode and to significantly increase the number of infective episodes in which a minimum of two blood culture sets are collected.

### Study Limitations

Our study has some limitations. First, it did not analyze the impact on the contamination reduction in each component of the bundle separately. Also, the follow-up period was 5 months, and we did not evaluate the long-term effect of our intervention, whether this downward trend will continue, and if a new intervention is necessary. This requires further evaluation.

## 4. Materials and Methods

The National Institute for Infectious Diseases “Prof. Dr. Matei Bals” Bucharest is a tertiary hospital, specializing exclusively in treating infectious diseases. It has only infectious diseases wards, 6 wards for adults, 5 wards for children, and 2 intensive care units. During 2023, 1 adult ward was closed for maintenance.

A blood culture set was defined as 2 blood culture bottles (one aerobic and one anaerobic) collected simultaneously. In the youngest pediatric population, due to their anatomical characteristics, 1 blood culture bottle was considered 1 set. A blood culture set was considered contaminated if it yielded well-known contaminants (CoN-staphylococci, *Corynebacterium* spp., *Micrococcus* spp., viridans group streptococci, *Cutibacterium* spp., *Bacillus* spp., etc.) in the absence of an immunosuppressed patient or a patient with indwelling devices.

An infectious episode with blood cultures was defined as an event produced by a single infection in which at least 1 blood culture set was collected.

The blood culture contamination rate was defined as the ratio between the number of contaminated blood cultures and the total number of blood culture samples expressed as a percentage.

Blood culture bottles (bioMérieux FA Plus, FN Plus, SA, SN, and PF plus, bioMérieux S.A., Marcy-l’Etoile, France) were incubated at 37 °C, and those that tested positive per the BacT/Alert (bioMérieux, Inc., Durham, NC, USA) were removed and processed by performing Gram staining and culture on Columbia sheep blood agar, chocolate agar, and lactose agar.

Plates were incubated in aerobic atmospheres at 35 °C ± 1 °C. Growth was observed at 18–24 h.

The microorganisms were identified using MALDI Biotyper^®^ (Bruker Daltonics GmbH & KG, Bremen, Germany).

### 4.1. Rationale of the Study

**Figure 4:**
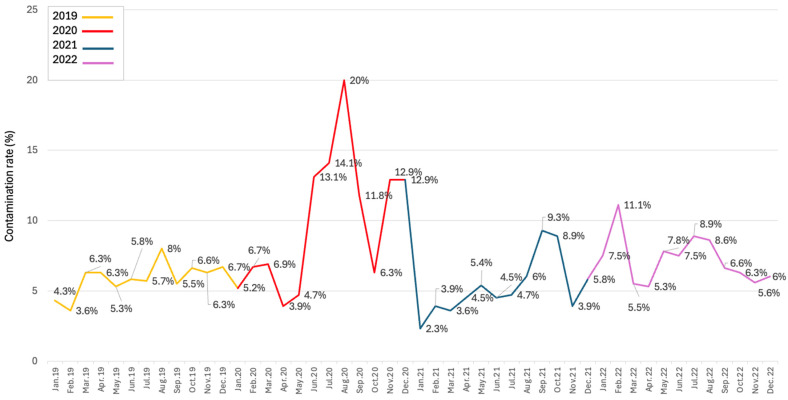
Evolution of the contamination rate during 2019–2022.

A retrospective analysis of the contamination rate in our hospital from 2019 to 2022 revealed an average contamination rate of 6.5% (Figure 4). In 2022, our contamination rate was 7.2%, far higher than the recommended rate, with almost half (47.4%) of the positive blood cultures being contaminated (unpublished data). This fact may be due to low adherence to our current blood culture collection protocol and nurse understaffing.

Also, the number of infectious episodes in which 2 or more blood culture sets were collected was low (837/2079 infectious episodes, 40.3%), with the clinicians ordering 1 single blood culture set/infectious episode in 59.7% (1242/2079 infectious episodes) of the cases (unpublished data).

Published analysis states that in order to increase the positivity rate, a minimum of 2 blood culture sets should be collected prior to starting antimicrobial therapy [33,34,35,36]. Baron et al. [36] stated that in order to optimize the use of blood cultures, 103–188 blood cultures/1000 patient days should be collected.

Currently, in our hospital, blood cultures are ordered by physicians (residents and senior physicians) and collected by nurses from each department. Alcohol and povidone-iodine for skin disinfection and sterile gloves for culture collection are recommended.

In this context, some interventions were needed to decrease the contamination rate of blood cultures and to increase the number of blood cultures sampled prior to antimicrobial therapy initiation.

### 4.2. Intervention Description

The intervention consisted of a discussion with the heads of the two departments over the results found on blood culture collection and contamination rates and by changing the current collection protocol. Also, we evaluated the nurses’ knowledge on blood culture collection with a questionnaire. It consisted of 12 questions, with 11 questions evaluating general knowledge on blood cultures and blood culture collection and 1 question revising the knowledge of the current protocol.

After revising the current recommendations and the literature articles on blood culture collection and interventions for decreasing the contamination rate, we decided to introduce a bundle of five elements:

- one-on-one discussions with the nurses about the new collection protocol, the changes made, and the reason behind them
- introducing 2% chlorhexidine in 70% alcohol for skin and top of the bottle disinfection
- ensuring the use of sterile gloves
- sterile wipes
- a checklist for the materials needed and the collection protocol available alongside the materials needed for blood culture collection

These changes did not add any significant costs to the hospital.

The intervention was conducted during July 2023, and the effect of these changes was observed for 5 months.

The study group consisted in the 2 adult wards: ward A (WA) and ward B (WB), and the control group consisted of the other 3 adult wards: ward C (WC), ward D (WD), and ward E (WE). We chose to perform the intervention in the 2 wards with the highest number of blood cultures/1000 patient days collected during January–July 2023 (120 blood cultures/1000 patient days in WA and 121.4 blood cultures/1000 patient days in WB, respectively, vs. 95.3/1000 patient days in WC, 103/1000 patient days in WD, and 114.4/1000 patients-days in WE, *p* < 0.0001).

The study’s data were extracted from the Bacteriology laboratory database and the before–after analysis was completed with Epi Info version 7.2.

Results were compared with data obtained from the other adult departments.

## 5. Conclusions

The five-bundle intervention decreased the contamination rate in the study group from 4% to 3.1% and the number of infectious episodes in which only a single blood culture set was collected before starting antimicrobial therapy.

## Figures and Tables

**Table 1:** Number of blood cultures collected/infectious episodes during January–December 2023.

	Blood Cultures Sets Collected	January–July 2023	August–December 2023	*p* (1-Side)
Control Group	one blood culture set (*n*; %)	376/895 (42%)	270/677 (39.9%)	*p* = 0.197
	≥2 blood culture sets (*n*; %)	519/895 (58%)	407/677 (60.1%)	*p* = 0.197
Study Group	one blood culture set (*n*; %)	139/456 (30.5%)	77/311 (24.8%)	*p* = 0.041
	≥2 blood culture sets (*n*; %)	317/456 (69.5%)	234/311 (75.2%)	*p* = 0.041

## Data Availability

Data are contained within the article.
